# Monocyte Chemoattractant Protein-1, a Possible Biomarker of Multiorgan Failure and Mortality in Ventilator-Associated Pneumonia

**DOI:** 10.3390/ijms20092218

**Published:** 2019-05-06

**Authors:** Yia-Ting Li, Yao-Chen Wang, Hsiang-Lin Lee, Su-Chin Tsao, Min-Chi Lu, Shun-Fa Yang

**Affiliations:** 1 Institute of Medicine, Chung Shan Medical University, Taichung 402, Taiwan; miki2700@gmail.com (Y.-T.L.); s31079@gmail.com (H.-L.L.); 2 Division of Respiratory Therapy, Department of Internal Medicine, Chung Shan Medical University Hospital, Taichung 402, Taiwan; 3 Division of Pulmonary Medicine, Department of Internal Medicine, Chung Shan Medical University Hospital, Taichung 402, Taiwan; wang5717@ms21.hinet.net; 4 School of Medicine, Chung Shan Medical University, Taichung 402, Taiwan; 5 Division of Gastroenterology, Department of Surgery, Chung Shan Medical University Hospital, Taichung 402, Taiwan; 6 Department of Nursing, Chung Shan Medical University Hospital, Taichung 402, Taiwan; suchin311@gmail.com; 7 Division of Infectious Diseases, Department of Internal Medicine, China Medical University Hospital, Taichung 404, Taiwan; 8 Department of Microbiology and Immunology, School of Medicine, China Medical University, Taichung 402, Taiwan; 9 Department of Medical Research, Chung Shan Medical University Hospital, Taichung 402, Taiwan

**Keywords:** ventilator-associated pneumonia, Monocyte chemoattractant protein-1, organ failure, mortality

## Abstract

Ventilator-associated pneumonia (VAP) leads to increased patients’ mortality and medical expenditure. Monocyte chemoattractant protein-1 (MCP-1) plays a role in the pathogenesis of lung inflammation and infection. Therefore, the plasma concentration of MCP-1 was assessed and correlated with the clinical course in VAP patients. This retrospective observational study recruited 45 healthy volunteers, 12 non-VAP subjects, and 30 VAP patients. The diagnostic criteria for VAP were based on the American Thoracic Society guidelines, and the level of plasma MCP-1 was determined by ELISA. Plasma MCP-1 concentration was significantly elevated in the acute stage in VAP patients when compared with the control (*p* < 0.0001) and non-VAP patient groups (*p* = 0.0006). Subsequently, it was remarkably decreased following antibiotic treatment. Moreover, plasma MCP-1 concentration was positively correlated with indices of pulmonary dysfunction, including the lung injury score (*p* = 0.02) and the oxygenation index (*p* = 0.02). When patients with VAP developed adult respiratory distress syndrome (ARDS), their plasma MCP-1 concentrations were significantly higher than those of patients who did not develop ARDS (*p* = 0.04). Moreover, plasma MCP-1 concentration was highly correlated with organ failure scores, including simplified acute physiology score II (SAPS II, *p* < 0.0001), sequential organ failure assessment score (SOFA, *p* < 0.0001), organ dysfunctions and/or infection (ODIN, *p* < 0.0001), predisposition, insult response and organ dysfunction (PIRO, *p* = 0.005), and immunodeficiency, blood pressure, multilobular infiltrates on chest radiograph, platelets and hospitalization 10 days before onset of VAP (IBMP-10, *p* = 0.004). Our results demonstrate that plasma MCP-1 is an excellent marker for recognizing VAP when the cut-off level is set to 347.18 ng/mL (area under the curve (AUC) = 0.936, 95% CI = 0.863–0.977). In conclusion, MCP-1 not only could be a biological marker related to pulmonary dysfunction, organ failure, and mortality in patients with VAP, but also could be used for early recognition of VAP.

## 1. Introduction

Ventilator-associated pneumonia (VAP) is a nosocomial pneumonia occurring more than 48 h after invasive mechanical ventilator support and is one of the most frequently diagnosed infectious diseases in the intensive care unit (ICU) [1,2]. Global incidence of VAP varies from 8 to 28% [3], and the development of VAP is associated with poor clinical outcome. VAP not only prolongs the duration of hospitalization but also entails considerable amount of healthcare costs. The mean hospital charge per VAP case has increased to nearly USD 40,000 [4,5,6] and is associated with a high mortality rate [7]. Therefore, stratifying mortality risk in patients with VAP and predicting the outcomes are important for making clinical decision and choosing treatment options when managing VAP [8].

Several scoring systems have been developed for critically ill patients, including simplified acute physiology score II (SAPS II) [9], sequential organ failure assessment score (SOFA) [10], organ dysfunctions and/or infection (ODIN) [11] and acute physiology and chronic health evaluation II (APACH II) [10]. The SAPS II contains 17 variables, including 12 physiologic parameters, age, type of admission, and 3 underlying conditions variables that were predictive of mortality using logistic regression. The SOFA score was developed to measure morbidity-associated dysfunction of six different organ systems [12]. The ODIN predicts the prognosis of critical patients based upon 12 variables of failure types derived from 6 organ systems and the infection status [13,14], and the APACHE II score provides a general measurement of disease severity employing 12 regular physiological factors, age, and previous health status as determined by the worst value during the initial 24 h of ICU admission [15,16]. They were also used to predict mortality risk in patients with VAP.

However, most of these scoring systems were not developed specifically for VAP, and only a few have been well validated in VAP [8]. Although APACH II has been well validated, its routine use in clinical practice is often arduous and burdensome. It contains a large number of components, and proper trained medical personnel is needed for accurate use [9,15]. Scoring systems were then developed specifically for VAP afterwards. The clinical pulmonary infection score (CPIS) is often used as a diagnostic tool for VAP; however, both its sensitivity and its specificity are unsatisfactory, particularly in predicting the severity of VAP. Thereafter, it was modified and used for guiding therapy [9,17,18,19,20]. Another scoring system based on the VAP PIRO (predisposition, insult response and organ dysfunction) concept and IBMP-10 (immunodeficiency, blood pressure, multilobular infiltrates on chest radiograph, platelets and hospitalization 10 days before onset of VAP) was developed. Unfortunately, these systems are still not satisfactory for either determining the severity or facilitating early recognition of VAP [21].

The inflammatory mediators chemokines are small proteins (8–10 kDa) categorized in the family of chemoattractant cytokines. They are involved in selectively recruiting monocytes, neutrophils, and lymphocytes. Chemokines also induce chemotaxis and play major roles in primary bacteria clearance [22,23,24,25]. Monocyte chemoattractant protein 1 (MCP-1) is a member of CC chemokines, also known as chemokines C–C motif ligand-2 (CCL2) [26]. They can be produced by various cells, including endothelial cells, fibroblasts, epithelial, smooth muscle, mesangial, astrocytic, monocytic, and microglial cells [22,27,28,29,30]. Above all, monocyte/macrophages are the major source of CCL2. During an inflammatory process, monocytes/macrophages phagocytize and remove pathogens in the involved tissue [22]. Therefore, MCP-1 plays an important role in routine immune surveillance and modulation, as well as in clearing acute bacterial burden [31]. In the pathogenesis of pneumonia, MCP-1 binds to its unique receptor chemokine C–C motif receptor (CCR2) to induce downstream signaling pathways [32,33] and has been demonstrated to be a powerful T cell and monocyte/macrophage chemoattractant against bacteria infecting the lung [34,35,36,37,38]. During *Streptococcus pneumonia* infection, CCL2-dependent lung exudate macrophages and conventional dendritic cell are recruited to contain the progression of pneumonia and contribute to lung protection [39]. In an in vivo study, Amano et al. found that MCP-1 promotes the resolution of inflammation and repairs the lung by augmenting the uptake of neutrophils recruited by alveolar macrophages following intrapulmonary *Pseudomonas aeruginosa* infection [33,40]. In addition, MCP-1 participates in adequate protective immune responses in pulmonary infection [25,41].

MCP-1 has been demonstrated to be deeply involved in the protection against bacterial infection and also participates in lung-protective immunity. Many previous studies examined the severity of pneumonia and discovered that it is both reflected and predicted by higher levels of cytokines in the blood [16,19,20,21,22]. For instance, in patients with community-acquired pneumonia [4], MCP-1 levels were shown to be correlated with disease severity. After reviewing these points, we hypothesized that MCP-1 could be a useful biomarker for both detecting the severity of VAP and facilitating the early recognition of VAP. To the best of our knowledge, there is no published study that has investigated the prognostic value of plasma MCP-1 in a cohort of patients with VAP. Herein, we compared plasma MCP-1 protein levels among groups of patients with clinical diagnosis of VAP (hereafter, patients with VAP), patients who received mechanical ventilation without subsequent VAP development (hereafter, patients without VAP), and healthy control volunteers, to find out whether if there is any association between plasma MCP-1 levels and VAP severity.

## 2. Results

In total, 87 patients were recruited for this study: 45 healthy volunteers, 12 patients without VAP, and 30 patients with VAP; their clinical and demographic characteristics are summarized in Table 1. Healthy volunteers were younger than both patients with VAP (36.53 ± 1.78 vs. 64.30 ± 3.19, *p* < 0.001) and patients without VAP (36.53 ± 1.78 vs. 52.67 ± 5.80, *p* = 0.01). Cigarette smoking was less frequent in healthy volunteers than in patients with VAP (*p* = 0.01; Table 1). Betal nut chewing was also less frequent in healthy volunteers than in patients without VAP (*p* = 0.02; Table 1). Moreover, the inflammation markers, including WBC and neutrophil levels (*p* < 0.001; Table 1) were significantly higher in patients with VAP. Surgical operation was the main reason for initiating ventilation support in patients without VAP (*p* = 0.02; Table 1).

We stratified the clinical scores into those that were developed non-specifically for VAP (APACH II, SAPS II, ODIN, SOFA, and Charlson comorbidity index) and those that were developed specifically for VAP (CPIS, PIRO, and IBMP10 concepts). They were used to recognize and predict mortality risk in patients with VAP (Table 2). The results showed that APACH II score, SAPS II, ODIN, SOFA, CPIS, PIRO, IBMP-10 scores (*p* < 0.0001), and Charlson comorbidity index (*p* = 0.01) were significantly higher in patients with VAP than in patients without VAP.

**Figure 1:**
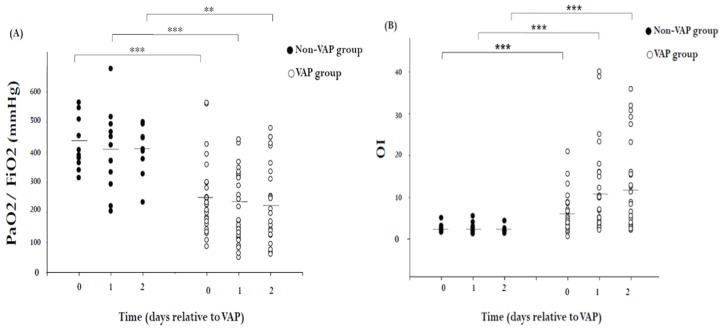
Scatter plots of respiratory data in 30 patients with VAP and 12 non-VAP subjects. (**A**) Hypoxemia score (the ratio of arterial oxygen partial pressure to fractional inspired oxygen; PaO2/FiO2) and (**B**) oxygenation index (OI = FiO2 × mean airway pressure/PaO2) in VAP patients and non-VAP patients (circle). Each dot represents a single patient. The solid lines mark the mean values. ** *p* < 0.01, *** *p* < 0.001 by Mann–Whitney *U* test.

Respiratory data are displayed in Figure 1. The hypoxemia score in patients without VAP was close to that of the control group. The hypoxemia score and OI were worse in VAP patients than in patients without VAP (*p* < 0.001). Microbiological data—stratified into high-risk (*Acinetobacter baumannii*, *P. aeruginosa*, *Stenotrophomonas maltophilia*, and *Staphylococcus aureus*) and low-risk bacteria [42]—from SQ-EA identification of VAP are shown in Table 3.

**Figure 2:**
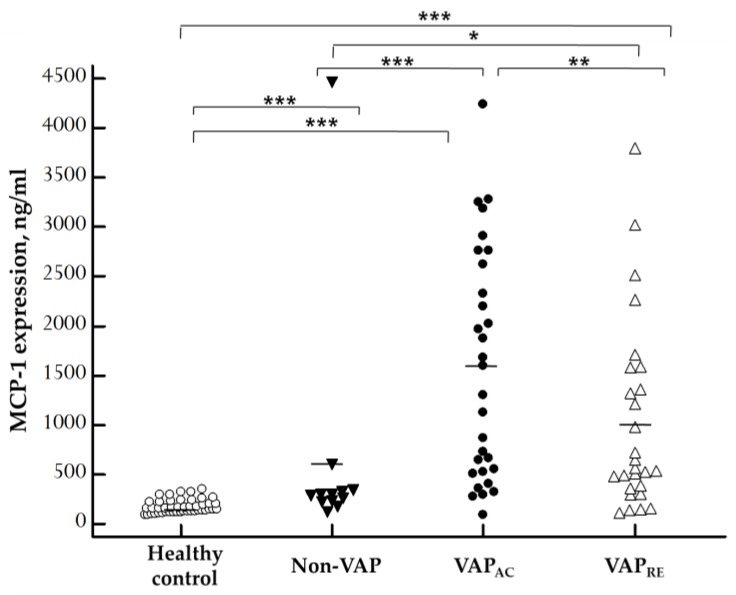
Scatter plots of plasma monocyte chemoattractant protein 1 (MCP-1) levels in 45 healthy control, 12 non-VAP subjects, and 30 patients with VAP who were divided into acute and remission stage according to ELISA results. MCP-1 concentration was significantly higher in the acute stage in VAP patients when compared with non-VAP subjects (*p* = 0.0006) and healthy controls (*p* < 0.0001), and remarkably decreased after treatment (*p* = 0.0082). Moreover, there was also significant difference between non-VAP subjects and healthy controls (*p* = 0.0007). Plasma MCP-1 levels were significantly increased in the remission stage in VAP patients when compared with non-VAP subjects (*p* = 0.0149) and healthy controls (*p* < 0.0001). Each dot represents a single patient. The solid lines mark the mean values. VAP_AC_, VAP in acute stage, VAP_RE_, VAP in remission stage. The Kruskal–Wallis test for multiple comparisons was used to determine the differences among the means of various plasma MCP-1, then the Mann–Whitney *U* test was employed to compare the results between groups, and the paired sample t-test was used for comparisons between acute and remission stage of VAP. * *p* < 0.05, ** *p* < 0.01, *** *p* < 0.001 by statistical analysis.

The concentrations of plasma MCP-1 in patients with VAP, patients without VAP, and the control group are displayed in Figure 2. Plasma MCP-1 levels were significantly increased in the acute stage in VAP patients (1579.72 ± 211.47 ng/mL) when compared with patients without VAP (632.86 ± 349.91 ng/mL; *p* = 0.0006) and the control group (176.02 ± 10.11 ng/mL; *p* < 0.0001). Plasma MCP-1 levels remarkably decreased after treatment (1027.09 ± 183.25 ng/mL; *p* = 0.0082). Furthermore, there was also significant difference between non-VAP subjects and healthy controls (*p* = 0.0007). Plasma MCP-1 levels were significantly increased even in the remission stage of VAP patients when compared with non-VAP subjects (*p* = 0.0149) and healthy controls (*p* < 0.0001).

**Figure 3:**
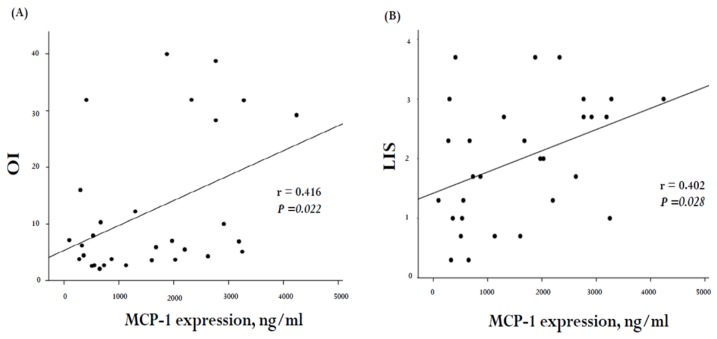
Correlation of plasma MCP-1 levels with severity of pulmonary dysfunction score in 30 VAP subjects. (**A**) There was a signification correlation between MCP-1 levels and OI (general linear model, *r* = 0.416, *p* = 0.022, *n* = 30) as well as (**B**) lung injury scores (LIS) (general linear model, *r* = 0.402, *p* = 0.028, *n* = 30).

In order to investigate the correlation between the severity of pulmonary dysfunction and MCP-1 levels in VAP patients, we adopted the OI and LIS as surrogates for the pulmonary dysfunction scores. The correlation between these scores and MCP-1 concentrations in our 30 VAP subjects is illustrated in Figure 3. A positive correlation was noted between MCP-1 levels and OI (*r* = 0.416, *p* = 0.022; Figure 3A) as well as LIS (*r* = 0.402, *p* = 0.028; Figure 3B).

**Figure 4:**
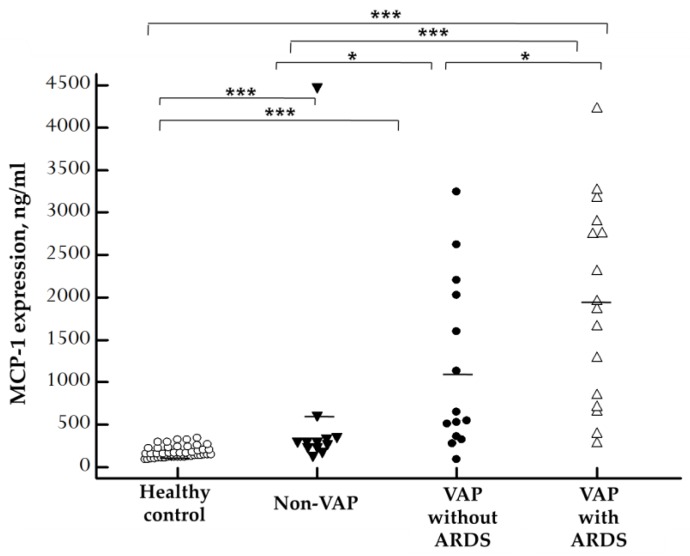
Scatter plots of plasma MCP-1 levels in 45 healthy control, 12 non-VAP subjects, and 30 patients with VAP who were divided into patients without ARDS and patients with ARDS on the basis of ELISA results. MCP-1 concentration was significantly higher in VAP-with-ARDS patients than in patients without ARDS (*p* = 0.0417), healthy controls (*p* < 0.0001), and non-VAP subjects (*p* = 0.0004). Moreover, there was a significant difference between non-VAP subjects and healthy controls (*p* = 0.0007). Plasma MCP-1 levels were significantly increased in VAP-without-ARDS subjects compared with non-VAP subjects (*p* = 0.0156) and healthy controls (*p* < 0.0001). Each dot represents a single patient. The solid lines mark the mean values. The mean concentration of plasma MCP-1 was determined by Kruskal–Wallis tests for multiple comparisons and then examined by the Mann–Whitney *U* test between groups. * *p* < 0.05, *** *p* < 0.001 by statistical analysis.

Since VAP can lead to ARDS, and there was a positive correlation between the plasma concentration of MCP-1 and pulmonary dysfunction scores, we hypothesized that the concentration of MCP-1 and ARDS could also be correlated. Therefore, we tried to explore the expression of MCP-1 in VAP patients who developed ARDS. The concentrations of plasma MCP-1 in VAP patients who developed ARDS, VAP patients without ARDS, and control group are displayed in Figure 4. We discovered that plasma MCP-1 levels were significantly increased in VAP patients who developed ARDS (1954.15 ± 294.81 ng/mL) when compared with patients without ARDS (1151.80 ± 269.73 ng/mL; *p* = 0.0417), healthy controls (176.02 ± 10.11 ng/mL; *p* < 0.0001), and non-VAP subjects (632.86 ± 349.91 ng/mL; *p* =0.0004). Moreover, there was a significant difference between non-VAP subjects and healthy controls (*p* = 0.0007). Plasma MCP-1 levels were significantly increased in VAP subjects without ARDS compared with non-VAP subjects (*p* = 0.0156) and healthy controls (*p* < 0.0001).

**Figure 5:**
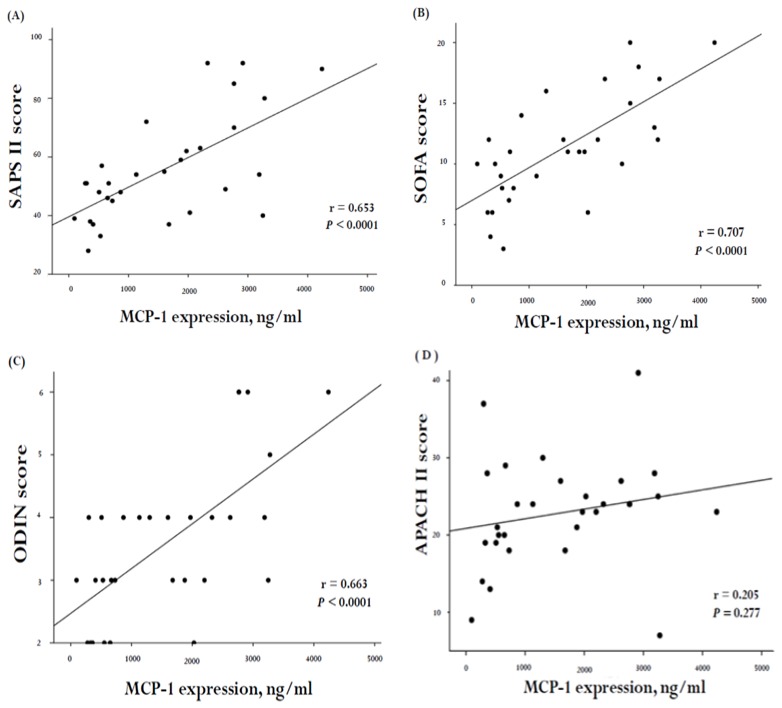
Correlation of plasma MCP-1 with severity of organ failure score (non-specifically for VAP) in 30 VAP subjects. There was a positive correlation between MCP-1 levels and (**A**) SAPSII (general linear model, *r* = 0.653, *p* < 0.0001, *n* = 30), (**B**) SOFA (general linear model, *r* = 0.707, *p* < 0.0001, *n* = 30), (**C**) ODIN (general linear model, *r* = 0.663, *p* < 0.0001, *n* = 30) and (**D**) APACH II (general linear model, *r* = 0.205, *p* *=* 0.277, *n* = 30)

**Figure 6:**
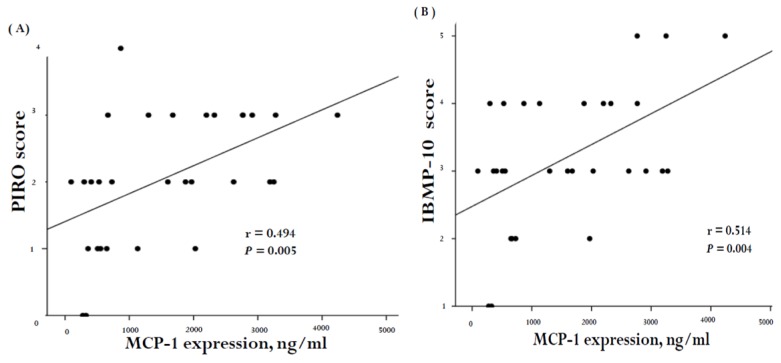

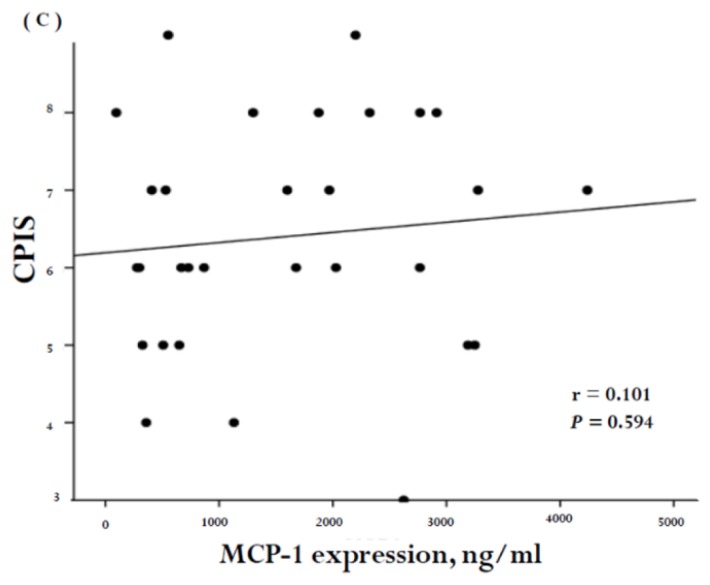
Correlation of plasma MCP-1 levels with severity of organ failure score (specifically for VAP) in 30 VAP subjects. Scoring system developed specifically for VAP, including (**A**) PIRO, (**B**) IBMP-10 concepts, and (**C**) CPIS. There was a significant correlation between MCP-1 levels and PIRO score (general linear model, *r* = 0.494, *p* = 0.005, *n* = 30) and IBMP score (general linear model, *r* = 0.514, *p* = 0.004, *n* = 30).

MCP-1 plasma concentration was significantly higher in patients who developed ARDS. This suggested that there was a correlation between MCP-1, disease severity, and organ dysfunction. In order to investigate whether a correlation existed, we stratified the clinical scores into those developed specifically for VAP (CPIS, PIRO, and IBMP-10 concepts) and those that were not VAP-specific (SAPS II, SOFA, ODIN, and APACH II score). The correlation between these scores and MCP-1 concentrations in the 30 VAP subjects is illustrated in Figure 5 and Figure 6. A positive correlation was noted between MCP-1 levels and the SAPS II score (*r* = 0.653, *p* < 0.0001; Figure 5A), SOFA (*r* = 0.707, *p* < 0.0001; Figure 5B), as well as the ODIN score (*r* = 0.663, *p* < 0.0001; Figure 5C). Furthermore, MCP-1 concentrations correlated positively with the PIRO (*r* = 0.494, *p* = 0.005; Figure 6A) and the IBMP-10 scores (*r* = 0.514, *p* = 0.004; Figure 6B).

**Figure 7:**
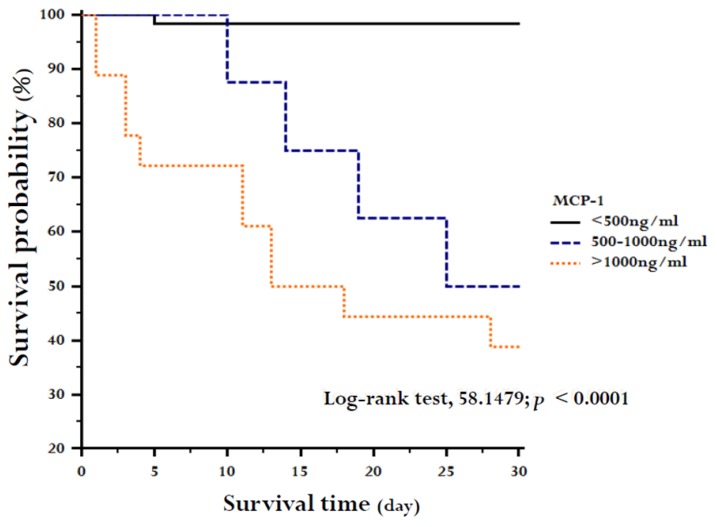
Kaplan–Meier estimates of survival during 28 days of study enrollment. The 87 subjects of the study were stratified into MCP-1 tertiles at enrollment.

When MCP-1 level was categorized in a unit of 500 ng/mL and VAP patients were divided in three categories with MCP-1 levels <500 ng/mL, 500–1000 ng/mL, and >1000ng/mL, MCP-1 tertiles at study enrollment differed significantly in survival (log-rank test, 58.1479; *p* < 0.0001; Figure 7).

**Figure 8:**
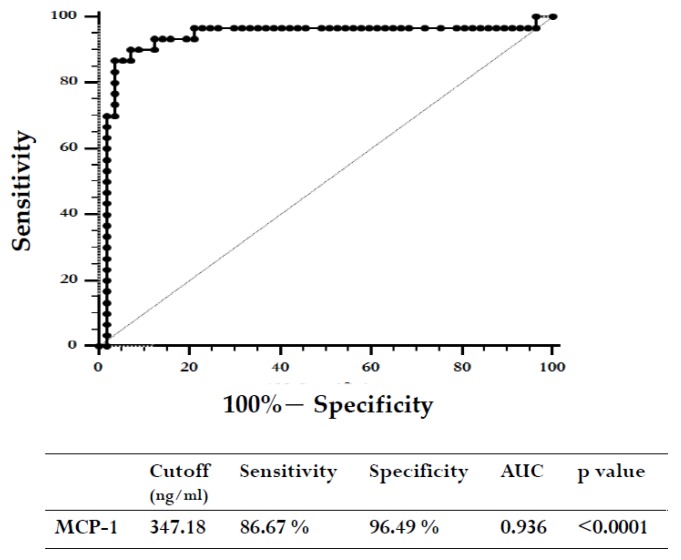
Receiver operating characteristic (ROC) curves for MCP-1 levels in the diagnosis of VAP.

Finally, we constructed ROC curves to determine the diagnostic accuracy of plasma MCP-1 levels for VAP. At cut-off level of 347.18 ng/mL, the area under the curve for MCP-1 was 0.936 (95% CI = 0.863 to 0.977; *p* < 0.0001) (Figure 8).

## 3. Discussion

Our study produced three major results. First, plasma MCP-1 were significantly elevated in the acute stage of patients with VAP when compared with both the control group and patients without VAP. MCP-1 decreased drastically after effective treatment. Besides, when ARDS developed, MCP-1 levels were significantly higher than in patient who did not develop ARDS. Second, plasma MCP-1 concentration was positively correlated with the pulmonary and organ dysfunction scores. Third, plasma MCP-1 concentration was an excellent biomarker for recognizing VAP and could potentially predict patients’ clinical outcome.

Napolitano et al. used logistic regression and identified that APACHE II score > 16 could be used as a predictor of ICU mortality during a VAP episode in pulmonary patients [8,27]. However, Thiago et al., using univariate analysis in 441 VAP patients, failed to identify a correlation between APACHE II score and mortality. Moreover, the authors constructed ROC curves to compare the severity assessment between VAP PIRO and APACHE II score, and the AUC (area under the ROC curve) showed consistent mortality discrimination by the VAP PIRO score (AUC = 0.81; 95% CI, 0.77 to 0.85), which outperformed the APACHE II score (AUC = 0.53; 95% CI, 0.47 to 0.58) [43]. In our study, we found that there was no correlation between MCP-1 levels and APACH II (general linear model, *r* = 0.205, *p* = 0.277) (Figure 5D). We attempted to investigate the cause of this and discovered that APACHE II scores were not computed on the day when VAP was diagnosed (APACHE II scores were determined by the worst value found during the initial 24 h after ICU admission). The time of APACHE II calculations varied widely and, in many cases, was longer than 10 days. Therefore, our results may not be able to detect the correlation between MCP-1 and APACH II. We tried to recalculate APACHE II score on the onset day of VAP but failed because this is a retrospectively designed study, and many data were missing. Additional studies are needed to develop and improve the practicability of APACHE II score in VAP groups.

Bernhard et al. described that high-risk pathogens seem to induce a strong neutrophil MMP (matrix metalloproteinase) release, which might be explained by the high virulence of these bacteria [42]. In our study, we tried to verify whether patients who were infected by high-risk or high-virulence bacteria could present a stronger host immune response leading to an increase in the concentration of MCP-1 and severe organ dysfunction. Our results show that there was no significant difference in the levels of MCP-1 between high- and low-risk bacterial infections (1530.60 ± 224.19 and 1899.05 ± 693.32) (*p* = 0.70) (data not shown). We speculate that, because most of our VAP patients were polymicrobial-infected (*n* = 25, 83.3%), it was difficult to detect the effect of high- and low-risk bacterial infections on the level of MCP-1. Therefore, future studies are needed to distinguish the relationship between MCP-1 and high- and low-risk bacteria that cause VAP.

In our study, we used three variables to detect the severity of pulmonary dysfunction, which were OI, LIS, and hypoxemia score. The OI is calculated by the following equation: OI = FiO2 × mean airway pressure/PaO2 and is described to be a measure for quantifying the severity of hypoxic respiratory failure [44]. The LIS was proposed by Murray and colleagues and is computed from four factors: (1) chest X-ray; (2) the degree of hypoxemia; (3) PEEP (positive end-expiratory pressure) level; and (4) static compliance. It has been a commonly utilized measure of acute lung injury severity in several clinical studies [9]. Hypoxemia score is expressed as PaO2/FiO2 (P/F ratio), is determined for acute hypoxic respiratory failure, and is one of the criteria for ALI (acute lung injury) or ARDS [45,46]. Our results showed that there was a positive correlation between OI and LIS on one hand and MCP-1 levels on the other (Figure 3). There was, however, no significant correlation between the hypoxemia score and MCP-1 levels (*r* = 0.337, *p* = 0.069). We noticed that the ventilator settings were not taken into the consideration to calculate the hypoxemia score, particularly the applied PEEP level. Optimal PEEP settings can improve oxygenation, especially in lung atelectasis [47,48,49]. Thus, the hypoxemia score may not be able to reflect the true severity of pulmonary dysfunction.

Bettina et al. displayed that the concentration of plasma MCP-1 elevation has the closest correlation with the development of pancreatic infections and with remote organ failure affecting the kidneys and the cardiocirculatory system as well [50]. Vermont et al. described that the levels of MCP-1 in patients with meningococcal sepsis are predictive of mortality and correlate best with disease severity [51]. Moreover, Fernando et al. reported that MCP-1 has good accuracy for predicting early (<48 h) and late (28-day) mortality in patients with sepsis [52]. In our study, we also discovered that plasma MCP-1 was significantly increased in VAP patients. It was positively correlated with disease severity and can potentially stratify patients with either good or poor clinical outcome. To the best of our knowledge, this is the first study that has successfully used MCP-1 as a biomarker in VAP patients. It could also be used for predicting disease severity and prognosis. Rau et al. described that chemokine activation may be considered as a potential link between the process of local injury, via induction of leukocyte infiltration, and the subsequent release of various inflammatory mediators leading to a systemic process involving distant organ systems [50]. Additional studies are required to discern the function of MCP-1 in local injury and distant organ failure.

There are several limitations in our study that should be mentioned. First, clinical severity was lower in the non-VAP group than in patients with VAP (Table 2). In the non-VAP group the duration of ICU stays and mechanical ventilation support was shorter, and the prescribed medication were fewer (data not shown). On the basis of this fact, Charlson comorbidity index was adopted for calculating comorbidity differences between the two groups. Our results showed that plasma MCP-1 levels were significantly increased in the acute stage in patients with VAP compared with patients without VAP (*p* = 0.005) (data not shown). Moreover, MCP-1 levels were still distinctly different (*p* = 0.007) (data not shown) even after confounding factors, including cancer (33.3% and 58.3% in VAP and non-VAP) and surgery (36.7% and 83.3%), were corrected (Table 1). Second, MCP-1 is associated with many pathological conditions [22], thus only non-hospitalized subjects were included in the healthy control group. The volunteers were younger (mean age <40 years) than the patients, and all study groups were predominantly composed of males. A statistic method was adopted to adjust for these confounders, and the results still showed significantly differences in the levels of MCP-1 (*p* = 0.004) (data not shown). However, this may still have led to a selection bias and represents a limitation of this study. Third, bias could also exist because the sample size in our study was small. Therefore, we propose that these results should be confirmed in a large-scale study.

## 4. Materials and Methods

### 4.1. Study Design and Definition

Among a total of 87 subjects from the same hospital, 45 were healthy volunteers, 12 patients went through surgeries (*n* = 10), had neurological conditions (*n* = 1) or cardiovascular failure (*n* = 1), and 30 patients suffered from VAP. All 45 healthy controls did not have cancers or inflammatory diseases. Patients were excluded if they were younger than 20 years of age at the time of study or had a coexisting extrapulmonary infection or immunosuppressive disease during the study period. This observational study was performed from June 2012 to June 2014 retrospectively in the intensive care units and respiratory care ward of Chung Shan Medical University Hospital (CSMUH) in Taichung, Taiwan.

The definition of VAP was based on the guidelines of the American Thoracic Society [53], including mechanical ventilation ≥ 48 h, new or developing infiltrates on chest X-ray, and at least two of the following: body temperature >38 °C or <36 °C, purulent tracheal secretions, and leukocyte count >10,000/μL or <4000/μL. In addition, an endotracheal aspirate with the characteristic of moderate growth and positive semiquantitative bacterial culture was a prerequisite for the condition to be categorized as VAP. The definition of adult respiratory distress syndrome (ARDS) was based on the guidelines of the Berlin definition. The diagnostic criteria included new or worsening respiratory symptoms over the last 7 days, bilateral opacities on chest radiographs, absence of suspected hydrostatic/cardiogenic pulmonary edema, and PaO2/FiO2 ≤ 300 with a PEEP level ≥ 5 cmH2O [47,54]. The study was approved by the Institutional Review Board of CSMUH (IRB No.CS13151, 29 October 2013), and informed consent was received from each participant.

### 4.2. Data and Blood Sample Collection

Patients’ baseline characteristics, namely, age, gender, history of cigarette smoking, drinking, betal chewing habit, inflammation markers including white blood cells (WBC), neutrophils, and C-reactive protein (CRP) levels, cause of respiratory failure, and coexisting illnesses were collected upon study enrollment. Clinical scores, including APACH II, SAPS II, ODIN, SOFA, comorbidity, CPIS, VAP PIRO concept, IBMP-10, and lung injury scores (LIS), respiratory parameters (including hypoxemia score; P/F ratio = PaO2/FiO2 [46,55], and oxygenation index, OI = FiO2 × mean airway pressure/PaO2), and microbiological data were recorded. Outcome assess was made at follow-up for 28 days or until patient’s death. Patients who died within 28 days after VAP onset were defined as non-survivors, all others were defined as survivors, and no patient was lost during the follow-up period.

Paired blood samples were collected after ≤5 days from VAP onset, defined as acute VAP stage, and within 10 ± 3 days from VAP onset, defined as remission VAP stage. For controls, blood samples from non-VAP patients were withdrawn 48 h after initiating ventilation support and at the onset of ARDS. All samples were EDTA-treated, centrifuged, and stored in a −80 °C freezer.

### 4.3. Measurement of Plasma MCP-1 Levels

The MCP-1 levels in the plasma samples were analyzed using human MCP-1 enzyme-linked immunosorbent assay (ELISA) kits (R&D Systems, Abingdon, UK) as previously described [56]. Briefly, the wells of an ELISA plate containing 0.1 mL of diluted samples were incubated at 37 °C for 2 h and, subsequently, 0.1 mL of anti-human MCP-1 antibody was added to each well. Each well was washed three times with 0.4 mL washing buffer, and then 0.2 mL of conjugate solution was added. Again, the wells were washed and loaded with 0.2 mL of TMB agent at 37 °C in a dark room for 30 min. For final processing, each well was filled with 0.1 mL of stop solution, and the amount of MCP-1 absorbance was quantitatively measured at 450 nm with a spectrophotometer and calibrated using human MCP-1 standards.

### 4.4. Statistical Analysis

Categorical variables were expressed as percentages, and the continuous value was expressed as the mean ± SE. Comparability of groups was analyzed by the Mann–Whitney *U* test, the *x*2 test, or the Fisher exact test, as suitable. To correct the possible confounding factors, including Charlson Comorbidity index, cancer, surgery, age, and gender, we transformed these numerical values to logarithms and adjusted them by the linear regression method. The statistical significance of the means for plasma MCP-1 was determined by the Kruskal–Wallis tests for multiple comparisons and then by the Mann–Whitney *U* test between groups and analyzed by paired sample *t*-test between acute and remission stage of VAP. A linear regression analysis was used to identify correlations between MCP-1 concentration and scores variables. Kaplan–Meier survival curves were employed to assess the time to death and were compared using the log-rank test. The estimation of cut-off concentrations was in accordance with their receiver operating characteristic (ROC) curves, and their identification employed the Youden index. Further examination of test performance was evaluated by Likelihood ratios. A *p* < 0.05 was considered statistically different by using the IBM SPSS statistics V20.0.0 software (SPSS, Chicago, IL, USA).

## 5. Conclusions

In conclusion, plasma MCP-1 concentration is correlated with the pathogenesis of VAP and the development of ARDS. MCP-1 not only could be a biological marker related to pulmonary dysfunction, organ failure, and mortality in patients with VAP, but also could be applied for early recognition of VAP.

## Figures and Tables

**Table 1:** Characteristics of ventilated patients and healthy control subjects.

	Patients with Ventilation		Controls
	Patients Developing VAP	Patients Not Developing VAP	
	(*n* = 30)	(*n* = 12)	(*n* = 45)
**Age (years)**	64.30 ± 3.19	52.67 ± 5.80	36.53 ± 1.78 ^a,b^
**Male gender**	25 (83.3%)	8 (66.7%)	39 (86.7%)
**History**			
Cigarette smoking	15 (50.0%)	4 (33.3%)	9 (20.0%) ^a^
Drink	7 (23.3%)	4 (33.3%)	8 (17.8%)
Betal	4 (13.3%)	5 (41.7%)	4 (8.9%) ^b^
**Inflammation markers at study enrollment**			
WBC (/mm^3^)	16,476.67 ± 1158.26	8350.00 ± 953.73 ^a^	7111.11 ± 315.67 ^a^
Neutrophils (/mm^3^)	13,285.40 ± 1088.20	5264.56 ± 898.17 ^a^	4188.40 ± 274.36 ^a^
CRP (ng/dL)	16.15 ± 1.69	-	-
**Reason for respiratory failure**			
Postoperative	11 (36.7%)	10 (83.3%) ^a^	
Lung disease	7 (23.3%)	0	
Neurologic failure	6 (20.0%)	0	
Trauma	1 (3.3%)	1 (8.3%)	
Sepsis	4 (13.3%)	0	
Shock	3 (10.0%)	0	
Cardiovascular	0	1 (8.3%)	
Miscellaneous	1 (3.3%)	0	
**Cormorbidities**			
CVA	4 (13.3%)	3 (25.0%)	
Cardiac	8 (26.7%)	3 (25.0%)	
Pulmonary	4 (13.3%)	1 (8.3%)	
Liver	3 (10.0%)	0	
Renal	4 (13.3%)	0	
Cancer	10 (33.3%)	7 (58.3%)	

WBC, white blood cell; CRP, C-reactive protein; CVA, cerebral vascular accident; VAP, ventilator-associated pneumonia; discrete variables are expressed as counts (%), and continuous variables as mean ± SE. ^a^ *p* value < 0.05 was considered significant in patients with VAP vs. non-VAP patients or healthy control subjects, ^b^ *p* value < 0.05 was considered significant in non-VAP patients vs. healthy control subjects.

**Table 2:** Clinical scores of patients with VAP and non-VAP subjects.

Characteristics	VAP (*n* = 30)	Non-VAP (*n* = 12)	*p* Value
**Non-specifically for VAP**			
APACH II	22.83 ± 1.29	14.00 ± 1.35	0.00
SAPS II	55.57 ± 3.27	25.50 ± 2.34	0.00
ODIN scores	3.60 ± 0.23	1.17 ± 0.11	0.00
SOFA scores	11.27 ± 0.81	4.00 ± 0.33	0.00
Comorbidity	6.23 ± 0.48	3.92 ± 0.66	0.01
**Specifically for VAP**			
CPIS	6.40 ± 0.27	1.58 ± 0.26	0.00
PIRO scores	2.07 ± 0.18	0.83 ± 0.11	0.00
IBMP-10 scores	3.20 ± 0.19	0.00 ± 0.00	0.00

Acute Physiology and Chronic Health Evaluation II (APACH II); SAPS II, simplified acute physiology score II; ODIN, organ dysfunctions and/or infection; SOFA, Sequential Organ Failure Assessment score; CPIS, Clinical Pulmonary Infection Score; continuous variables as mean ± SE, *p* value < 0.05 was considered significant.

**Table 3:** Isolated pathogens in patients with VAP.

Bacteria	Number of Patients
**High-risk bacteria**	
*Acinetobacter baumannii*	14 (46.7%)
*Pseudomonas aeruginosa*	13 (43.3%)
*Stenotrophomona maltophilia*	11 (36.7%)
*Staphylococcus aureus*	3 (10.0%)
**Low-risk bacteria**	
*Enterobacter aerogenes*	6 (20.0%)
*Elizabethkingia meningoseptica*	5 (16.7%)
*Klebsiella pneumoniae*	4 (13.3%)
*Sphingomonas paucimobilis*	2 (6.7%)
*Escherichia coli*	1 (3.3%)
*Serratia marcescens*	1 (3.3%)
*Citrobacter koseri*	1 (3.3%)
*Proteus mirabilis*	1 (3.3%)
*Morganella*	1 (3.3%)

Data are expressed as number (percentage).

## Abbreviations

APACH II	Acute Physiology and Chronic Health Evaluation II
ARDS	adult respiratory distress syndrome
AUC	area under the ROC curve
CCL2	chemokine C–C motif ligand-2
CCR2	chemokine C–C motif receptor-2
CPIS	clinical pulmonary infection score
CRP	C-reactive protein
CVA	cerebral vascular accident
ELISA	enzyme-linked immunosorbent assay
ICU	intensive care unit
LIS	lung injury scores
MCP-1	monocyte chemoattractant protein-1
MMP	matrix metalloproteinase
ODIN	organ dysfunctions and/or infection
OI	oxygenation index
PEEP	positive end-expiratory pressure
P/F ration	PaO2/FiO2 ration
ROC	receiver operating characteristic
SAPS II	simplified acute physiology score II
SOFA	Sequential Organ Failure Assessment score
SQ-EA	semiquantitative endotracheal aspirates
VAP	ventilator-associated pneumonia
WBC	white blood cells
